# Leukemic presentation of ALK-positive anaplastic large cell lymphoma in an adult patient with hemophagocytic lymphohistiocytosis

**DOI:** 10.1007/s12308-026-00724-3

**Published:** 2026-07-30

**Authors:** Mark Oliver, Jenny Byrd, Eduard Matkovic

**Affiliations:** School of Medicine and Public Health, Department of Pathology & Laboratory Medicine, University of Wisconsin-Madison, Madison, WI USA

**Keywords:** Anaplastic large cell lymphoma, Leukemic phase, ALK-positive, Peripheral blood involvement, Hemophagocytic lymphohistiocytosis, Flow cytometry

## Abstract

Systemic ALK + anaplastic large cell lymphoma (ALCL) is a CD30-positive T cell neoplasm that commonly affects lymph nodes with frequent involvement of extranodal sites. A leukemic phase with peripheral blood involvement is extremely rare, and therefore, recognition of this uncommon phenomenon is crucial to avoid misdiagnosis and delayed treatment. We report a case of a previously healthy adult patient who presented with peripheralizing ALK-positive ALCL accompanied by hemophagocytic lymphohistiocytosis (HLH). Comprehensive ancillary studies were essential for establishing the diagnosis and determining blood involvement.

## Introduction

Anaplastic large cell lymphoma (ALCL) is a mature CD30-positive T cell neoplasm that comprises approximately 5% of all non-Hodgkin lymphomas [1]. Characterized by pleomorphic cells with abundant cytoplasm and distinct horseshoe-shaped nuclei (“hallmark cells”), ALCL commonly shows loss of one or more T cell markers. Based on expression or rearrangement of anaplastic lymphoma kinase (ALK), ALCL is subclassified into ALK-positive and ALK-negative disease, with each subtype showing distinct epidemiologic and prognostic characteristics. Most patients with ALCL present with advanced stage disease with peripheral or abdominal lymphadenopathy, frequently associated with extranodal infiltrates in skin, bone, soft tissue, and lungs [1–3].

Peripheral blood involvement is rare in ALCL. Among patients with leukemic-phase ALCL, ALK-positive cases are more common, and patients tend to be younger, predominantly male, with higher rates of small cell morphology and poor response to chemotherapy [4–8]. While long-term survival is significantly better in nonleukemic ALK + ALCL compared to ALK-negative ALCL, both subtypes show uniformly poor outcomes once circulating disease develops [4–10]. Hemophagocytic lymphohistiocytosis (HLH) is a clinically significant complication of ALCL; however, HLH onset is not typically reported at initial presentation of leukemic-phase ALCL.

We report a case of peripheralizing ALK-positive ALCL in an adult male. Notably, this case exhibited features of the common pattern with circulating large cells, rather than the more frequently reported small cell variant associated with peripheral blood involvement. The atypical presentation with HLH complicated the initial diagnosis and underscores the importance of recognizing circulating ALCL, as well as performing a thorough evaluation for an underlying T cell neoplasm in the differential diagnosis of HLH.

## Clinical history

A 65-year-old man with a history of hypertension was found collapsed and transported by EMS to an outside hospital with weakness, fever, and unintentional weight loss. He was hypotensive and tachycardic with acute kidney injury (creatinine 2.63 mg/dL). CBC showed anemia, thrombocytopenia, and leukocytosis, and the outside peripheral blood differential reported increased monocytes. Initial concern was for sepsis; however, CT imaging demonstrated splenomegaly and extensive lymphadenopathy above and below the diaphragm. Lymph node and bone marrow biopsies were performed, and peripheral blood was sent to a regional referral center for further evaluation.

Subsequent pathologic evaluation established a diagnosis of ALK-positive ALCL involving the lymph node, bone marrow, and peripheral blood. Additional laboratory studies were remarkable for elevated ferritin (12,787 ng/mL), soluble interleukin-2 receptor (297,272 pg/mL), and hypertriglyceridemia (455 mg/dL). The patient fulfilled 7 of 8 HLH-2004 criteria: fever, splenomegaly, bicytopenia, hypertriglyceridemia, hemophagocytosis, hyperferritinemia, and elevated soluble interleukin-2 receptor.

### Treatment and outcome

The patient received etoposide and dexamethasone for lymphoma-associated HLH, followed by brentuximab vedotin plus cyclophosphamide, doxorubicin, and prednisone (BV-CHP) for ALCL. He relapsed 10 months later with pulmonary, osseous, and nodal disease and subsequently responded to second-line crizotinib.

## Materials and methods

The CBC was performed on a Sysmex XN-9100 automated hematology analyzer along with a manual differential. Peripheral blood and bone marrow aspirate smears for review were stained with Wright stain. Flow cytometric analysis was carried out using stain-lyse-wash standard sample preparation protocol. Three tubes containing up to 10-color antibody combinations, including stacked markers, were used; configurations are shown in Table 1. Data was acquired using FACSCanto™II Clinical Flow Cytometry System with the FACSDiva™ Software. Immunohistochemistry was performed using BenchMark ULTRA PLUS system under routine Clinical Laboratory Improvement Amendments (CLIA)–compliant conditions. ALK immunohistochemistry using clone ALK1 was performed on the lymph node and bone marrow specimens. Interphase FISH was performed using ALK (2p23) break-apart probe (performed at OHSU Knight Diagnostic Laboratories). HTLV-1/2 serology and the following HLH-associated laboratory studies were tested: ferritin, triglycerides, fibrinogen, coagulation studies, liver chemistries, and soluble IL-2 receptor.

**Table 1:** Flow cytometry antibody panels used for immunophenotypic analysis of peripheral blood. The table lists antibody combinations, fluorochromes, and markers included in each tube

Tube	FITC	PE	PerCP-Cy5.5	PE-Cy7	APC	APC-H7	BV421	V500-C
Tube 1	sKappa and CD8	sLambda	CD4	CD10	CD19	CD3 and CD20	CD5	CD45
Tube 2	CD57	CD7	CD16	CD56	CD8	CD3	CD2	CD45
Tube 3	CD26	CD7	CD4	CD3	CD8	CD45	CD2	––

## Results

### Peripheral blood findings, flow cytometry, and ALK FISH

**Fig. 1:**
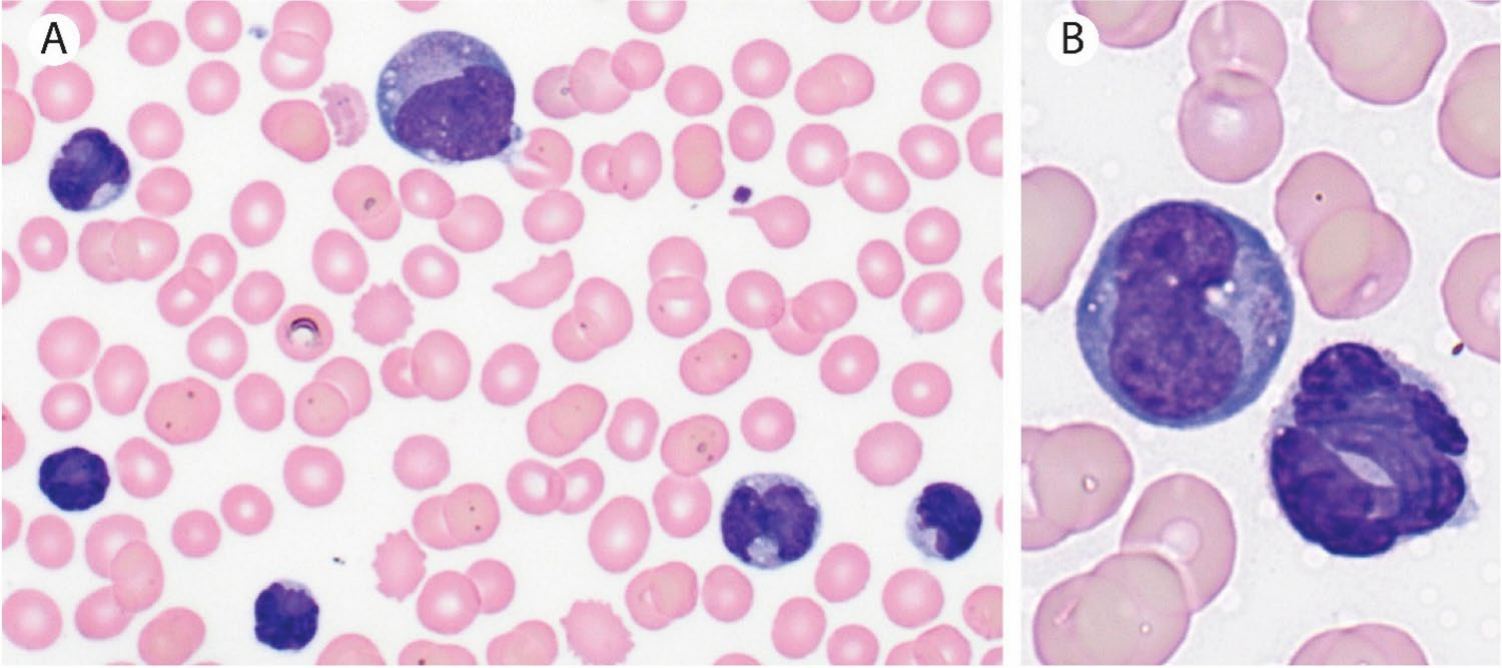
Peripheral blood smear microscopy and flow cytometry findings. **A** Peripheral blood smear demonstrates large atypical lymphocytes, 40 × objective (Wright stain). **B** The smear demonstrates pleomorphic cells with horseshoe-shaped nuclei, 100 × objective

**Fig. 2:**
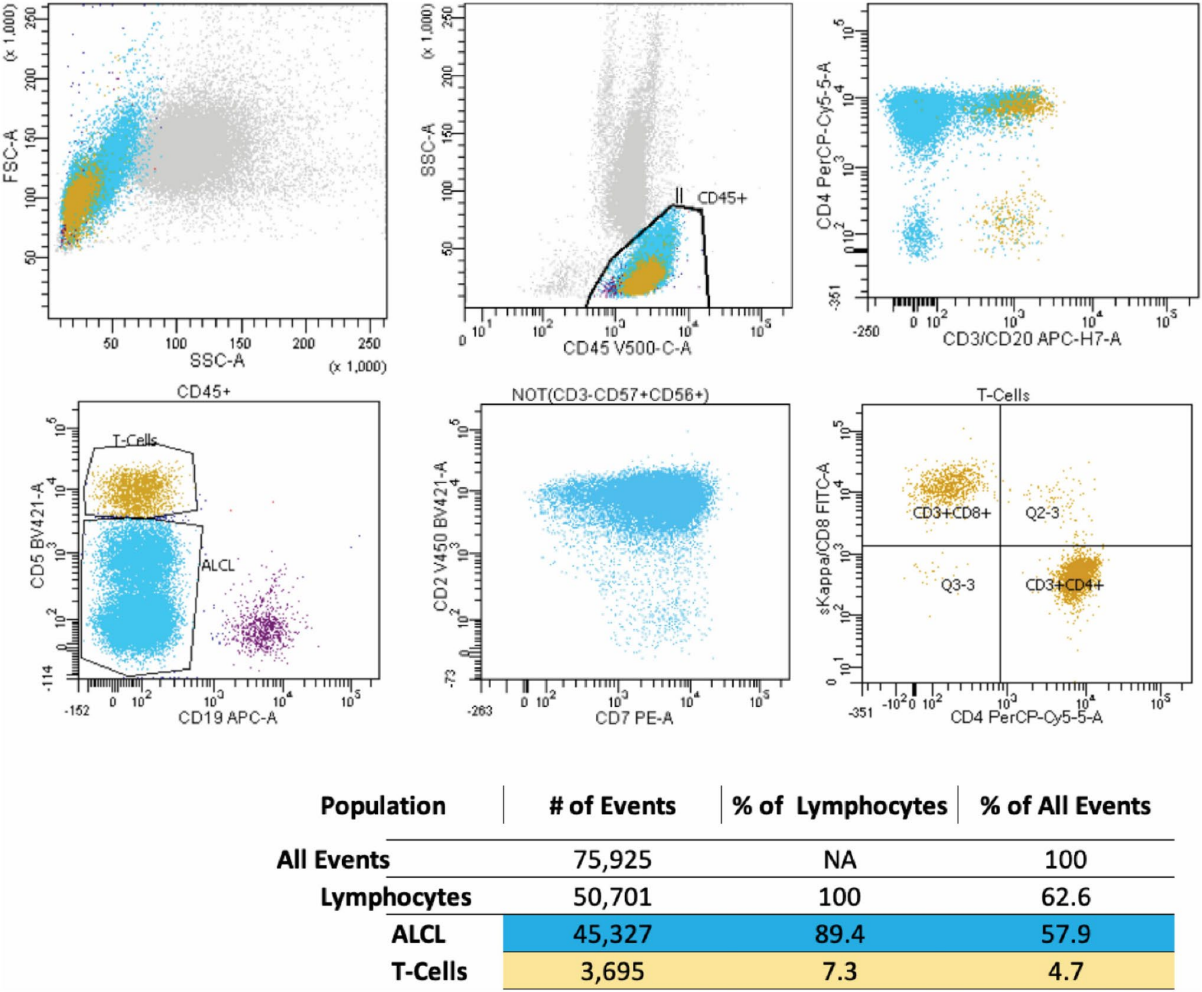
Flow cytometry findings. Flow cytometry demonstrates a neoplastic population, painted blue, of medium-to-large CD4-positive T cells with loss of CD3 and CD5 expression and positive for CD2 and CD7. Normal (reactive) T cells are painted yellow. Monocytes were scant and did not interfere with the analysis

Our CBC showed hemoglobin 6.7 g/dL, platelet count of 17 K/µL, and white blood cell count of 63 K/µL with 73% lymphocytes, 22% neutrophils, and 5% monocytes on the differential. Peripheral blood smear showed an increased number of large atypical lymphoid cells, but unlike the outside differential, only few monocytes were present (Fig. 1A, B). Lymphoid cells were mostly medium-to-large in size with ample basophilic cytoplasm and irregular or folded nuclei; some of which showed classic “hallmark” forms with horseshoe- or kidney-shaped nuclei. Flow cytometry was performed on the blood sample, and most lymphocytes identified by CD45 vs side scatter (SSC) were abnormal T cells positive for CD45, CD4, CD2, and CD7 and negative for surface CD3, CD5, CD8, CD56, and CD57 (Fig. 2). The lymphocytes showed increased SSC properties and extended into the monocyte region. The remaining blood sample was sent for ALK rearrangement by FISH, which confirmed peripheral blood involvement by ALCL. While abnormal T cells made up 89% of lymphocytes and 58% of all events on flow, only low-level ALK rearrangement was found in the blood by FISH, with split signal pattern observed in 9/200 (4.5%) cells, above the normal cutoff (≤ 4%).

### Lymph node biopsy and immunohistochemistry

**Fig. 3:**
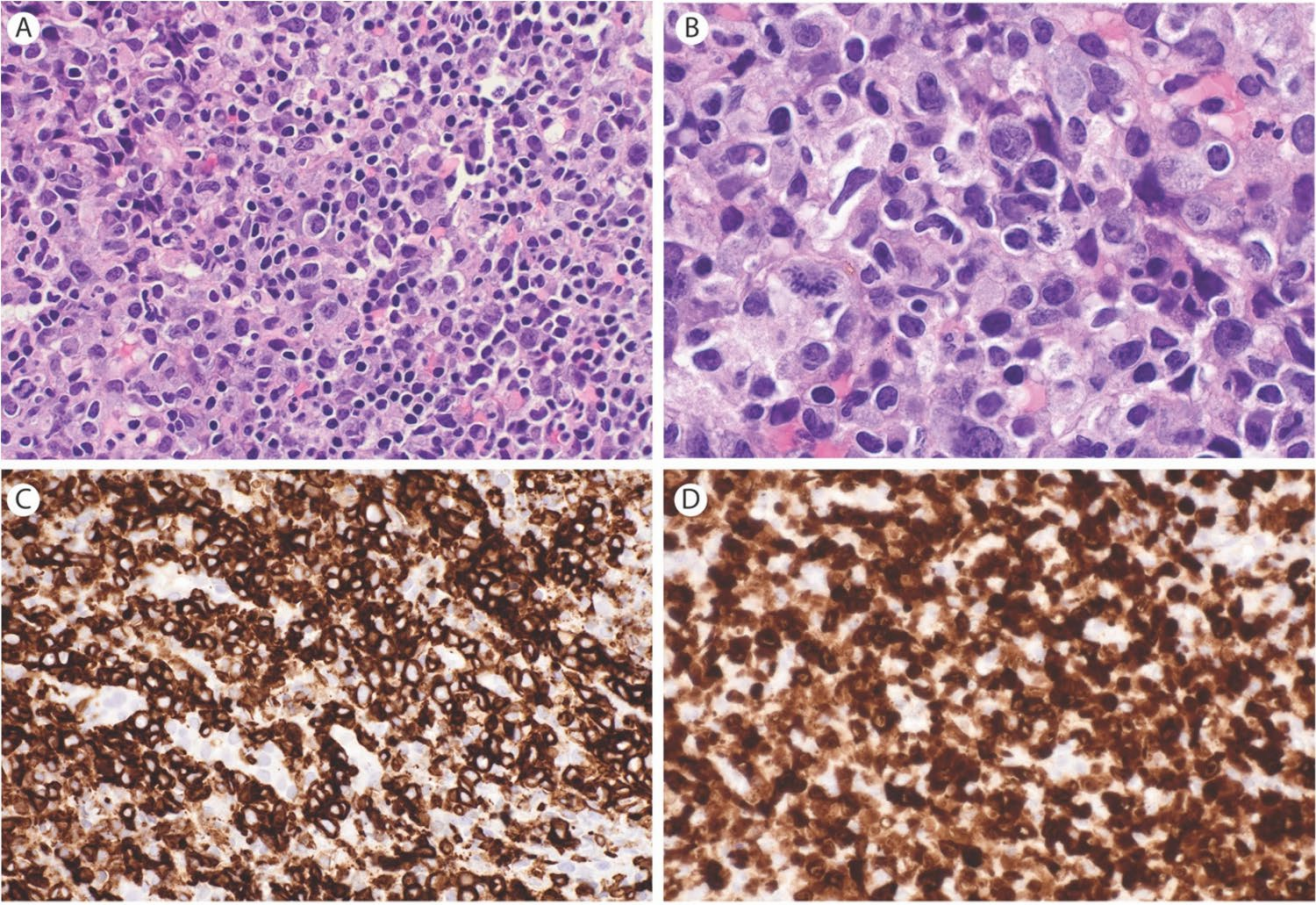
Lymph node microscopy and immunohistochemistry. **A** Peripheral lymph node, H&E stain, 20 × objective. **B** Peripheral lymph node demonstrating anaplastic lymphocytes, 40 × objective. **C** Lymph node with CD30 immunohistochemistry, 20 × objective. **D** Lymph node with ALK immunohistochemistry, 20 × objective

The lymph node needle core biopsy showed partial nodal effacement from confluent aggregates of atypical lymphoid cells (Fig. 3A–D). Aggregates contained a variable number of large cells with abundant cytoplasm and eccentric horseshoe-shaped nuclei mixed with smaller cells with irregular nuclear contours. Neoplastic cells demonstrated an immunophenotype concordant with the large pleomorphic cells in the peripheral blood and bone marrow, with strong CD30 and ALK expression by immunohistochemistry. The nuclear, nucleolar, and cytoplasmic ALK staining pattern was suggestive of an NPM1::ALK fusion; however, a molecular technique to identify the fusion partner was not performed.

### Bone marrow biopsy

**Fig. 4:**
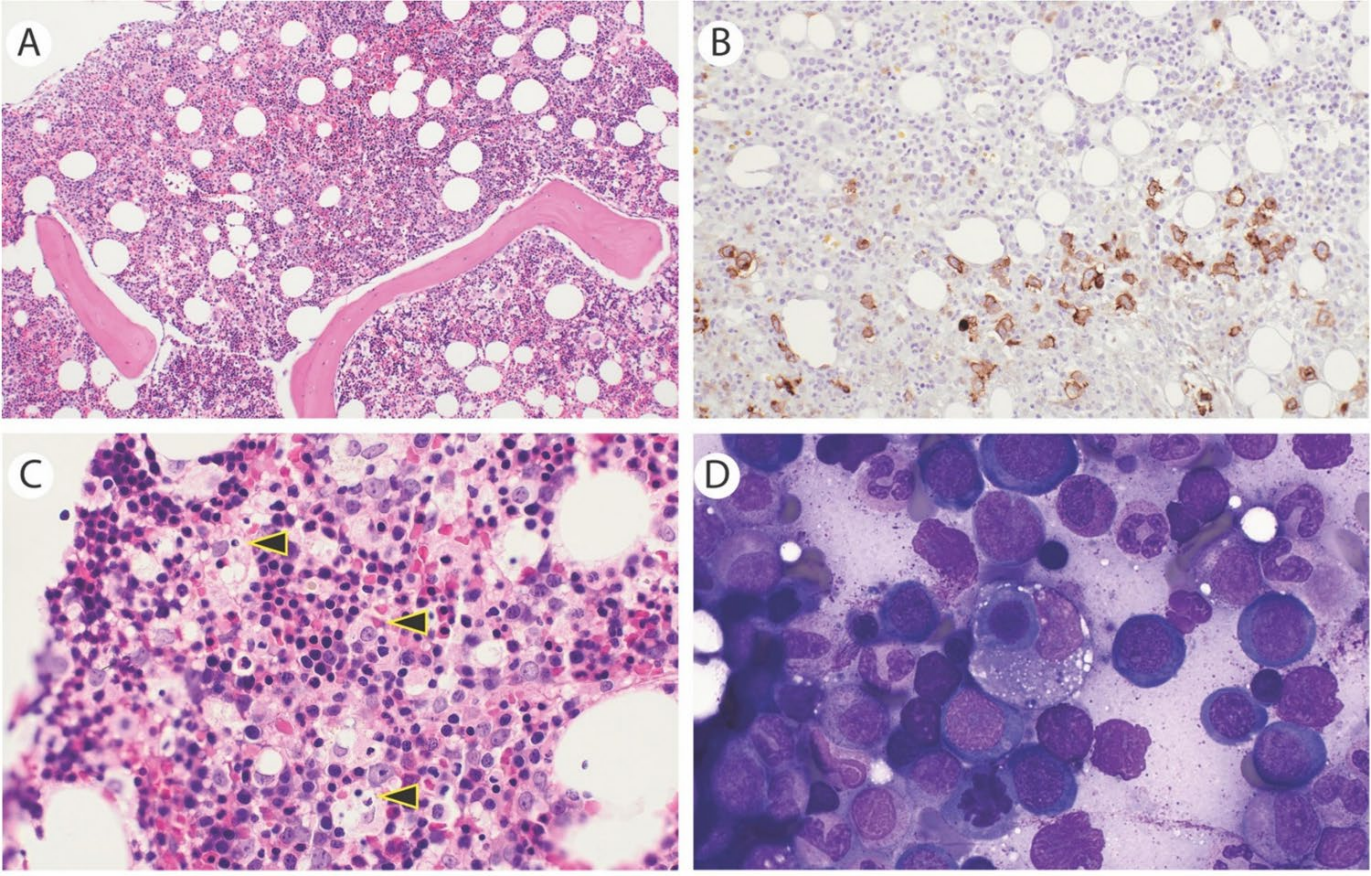
Bone marrow biopsy and smear. **A** H&E stain demonstrates hypercellular bone marrow, 10 × objective. **B** Minimal marrow involvement highlighted by CD30 immunohistochemistry, 20 × objective. **C** Core biopsy demonstrates phagocytosed erythroid and leukocyte precursors (arrowheads), 40 × objective. **D** Bone marrow aspirate demonstrating phagocytosed erythroid precursor, 100 × objective

Bone marrow core biopsy was hypercellular with trilineage hematopoiesis. There was minimal marrow involvement by lymphoma determined primarily by flow and CD30 IHC labeling a few large cells (Fig. 4A–D). Notably, the aspirate smears showed increase in macrophages with phagocytosed blood cells within their cytoplasm, including erythroid precursors and leukocytes. Core biopsy reflected the aspirate showing an interstitial distribution of macrophages with hemophagocytic activity on H&E and by CD68.

## Discussion

Leukemic-phase ALCL is rare and often occurs in younger patients with ALK rearrangement, circulating “small” lymphoma cells, and marrow involvement [4, 5 ]. Our patient had an ALK rearrangement but was an adult with large circulating lymphoma cells, including hallmark cells. The lymph node showed a common morphologic pattern with large pleomorphic cells, reflecting the peripheral blood. Circulating ALCL is a recognized clinicopathologic pitfall; our case initially mimicked sepsis with fever, hypotension, and renal dysfunction. Monocytosis was first reported on the outside white cell differential. Flow cytometry was also performed at the outside institution but did not detect the abnormal T cell population. The original dot plots were not reviewed at our hospital, but ALCL may fall in the monocyte region on CD45/SSC plots and may be missed with conventional lymphocyte gating [11]. Our flow analysis identified abnormal CD4-positive T cells when the lymphocyte gate was extended into the monocyte region. Along with hallmark cells on the blood smear, these findings were key to suggesting blood involvement before tissue diagnosis. Because surface CD30 was not included in our T cell flow panel, we ordered ALK FISH. Since circulating malignant cells can be difficult to distinguish from other large cell lymphomas, adding CD30 to the panel can aid diagnosis, and we have since incorporated this marker into our tube.

ALCL is a T cell lymphoma characterized by strong, uniform CD30 expression with variable T cell and cytotoxic marker expression. Our case showed aberrant surface CD3 and CD5 expression but was positive for CD2 and CD7. Loss of surface CD3 is not unusual in ALCL, and retained CD7 is common in leukemic phase of ALK-negative ALCL and in adults with ALK + anaplastic large cell lymphoma, small cell variant [5, 8, 12 ]. Neoplastic T cells made up 89% of blood lymphocytes by flow, and low-level ALK rearrangement (4.5%) exceeded the normal cutoff, supporting circulating disease. In any case, the diagnosis was established on the lymph node biopsy. Although an exact ALK fusion pattern was not identified, the ALK immunostaining pattern was informative because it may reflect the underlying fusion partner. Our ALK IHC showed the characteristic nuclear, nucleolar, and cytoplasmic staining pattern most often associated with NPM1::ALK [13].

HLH is not commonly reported in leukemic-phase ALCL and may dominate the clinical presentation before the lymphoma is obvious [14]. Severe HLH can occur despite minimal marrow involvement by lymphoma, highlighting the need to consider lymphoma-associated HLH. ALK-positive systemic ALCL generally responds better than ALK-negative disease, but leukemic-phase presentation and HLH both signal high-risk biology and severe illness. Early relapse with lung, bone, and nodal disease underscores that even ALK-positive disease can behave aggressively in this setting.

This case highlights a rare leukemic-phase presentation of ALK-positive systemic anaplastic large cell lymphoma complicated by HLH. It illustrates the importance of integrating peripheral blood morphology, flow cytometry, tissue biopsy for rapid diagnosis, and cytogenetic studies when evaluating a sepsis-like illness with cytopenias, marked leukocytosis, and very high ferritin. Awareness of this entity may help avoid diagnostic delay, prevent misclassification as a reactive process, and facilitate timely initiation of appropriate therapy.

## Data Availability

No datasets were generated or analyzed during the current study.
